# Lower-limb intramedullary nailing in patients with polyostotic fibrous dysplasia who had a previous unsuccessful treatment. A report of 48 cases

**DOI:** 10.1186/s10195-023-00705-7

**Published:** 2023-07-04

**Authors:** E. Ippolito, P. Farsetti, R. Caterini, G. Gorgolini, A. Caterini, F. De Maio

**Affiliations:** Department of Clinical Science and Traslational Medicine, Section of Orthopaedics and Traumatology, University of Rome “Tor Vergata”, Viale Oxford 81, 00133 Rome, Italy

**Keywords:** Polyostotic fibrous dysplasia, McCune–Albright syndrome, Lower limb fracture, Lower limb deformity, Salvage intramedullary nailing

## Abstract

**Background:**

Intramedullary nailing (IN) seems to be the best primary surgical treatment for patients with either polyostotic fibrous dysplasia or McCune–Albright syndrome (PFD/MAS) when the femur and tibia are totally affected by fibrous dysplasia (FD) and pain, fracture and deformity are likely to occur. However, other management protocols have been applied in these cases, often leading to disabling sequelae. This study sought to evaluate if IN could also have been effective as a salvage procedure to provide patients with satisfactory results, regardless of the poor results due to the improper treatment previously performed.

**Materials and methods:**

Twenty-four retrospectively registered PFD/MAS patients with 34 femurs and 14 tibias totally affected by fibrous dysplasia had received various treatments with unsatisfactory results in other institutions. Before the IN performed in our hospital, 3 patients were wheelchair bound; 4 were fractured; 17 limped; and many used an aid for walking. Salvage IN was performed in our hospital at a mean patient age of 23.66 ± 6.06 years (range, 15–37 years). The patients were evaluated before—except for the four fractured ones—and after IN using the validated Jung scoring system, and the data were statistically analyzed.

**Results:**

The mean length of follow-up after IN was 9.12 ± 3.68 years (range, 4–17 years). The patients’ mean Jung score significantly improved from 2.52 ± 1.74 points before IN to 6.78 ± 2.23 at follow-up (*p* < 0.05). Ambulation was improved in ambulatory patients and restored in wheelchair users. The complication rate was 21%.

**Conclusions:**

Regardless of the high rate of complications, IN may be considered a reliable surgical procedure to salvage a failed treatment in PFD/MAS, with long-lasting satisfactory results achieved in most patients.

*Trial registration statement*: Not applicable.

*Level of evidence*: IV.

## Introduction

PFD/MAS is a rare disease characterized by skeletal lesions, hyperfunctioning endocrinopathies and skin hyperpigmentation (cafè-au-lait macules). Its estimated prevalence is between 1/100,000 and 1/1,000,000. The disease is caused by somatic mutations of the GNAS gene, leading to an increased synthesis of the cAMP-regulating protein Gs alpha. The latter upregulates the proliferation of osteoblasts that are not able to reach their mature stage, leading to the formation of abnormal bone tissue with the histologic features of fibrous dysplasia. Gs alpha protein also upregulates the hormonal synthesis of one or more endocrine glands, with the consequent clinical manifestations. The diagnosis of PFD/MAS can be made on clinical grounds. Radiology and bone scans are useful for assessing bone lesions, while hormonal assays are appropriate for screening endocrinopathies. Usually, the first clinical manifestations of the disease are precocious puberty and femoral fracture or deformity [1].

In polyostotic fibrous dysplasia (PFD) and McCune–Albright syndrome (MAS), when the femur and tibia are totally affected, pain, fracture and deformity frequently occur, and patients may become disabled if the treatment is not appropriate [2–15].

Non-operative orthopedic management should be abandoned because its results are disappointing, and there is agreement nowadays that PFD/MAS patients require surgical treatment, particularly when the lower limb is extensively involved [4, 6, 7, 12, 13, 16–20]. However, not all the surgical options produce satisfactory results; for instance, peripheral plating is still performed in the lower limbs [18–25] regardless of the high incidence of complications reported by many authors [3, 9–17, 26, 27]. Intramedullary nailing (IN) of the femur or tibia seems to be the best primary surgical treatment available to obtain satisfactory results. However, the femur must be fixed with a cervicodiaphyseal nail to prevent varus deformity of the femoral neck, which is otherwise likely to occur if only the femoral shaft is nailed [3, 9–13, 15, 17, 18, 26, 27].

The aim of our study was to verify if IN could also have been effective in PFD/MAS patients who were variously treated elsewhere with unsatisfactory results.

## Materials and methods

After institutional review board approval was obtained, 29 consecutive patients with either PFD or MAS operated on by performing femoral and tibial IN—following unsuccessful treatments performed in other hospitals—were identified from the registry of our operating room between January 1999 and December 2016. One patient had had massive perioperative bleeding after his first femoral IN. He was excluded from the study since he refused any further treatment. Two other patients were excluded because their femurs and tibias were only partially affected by fibrous dysplasia (FD), while 2 patients refused to participate. The remaining 24 patients with their femurs and tibias totally affected by biopsy-proven FD gave us their informed consent and were enrolled in our study. The demographics, previous treatments and FD involvement of our patients are reported in Table 1.

**Table 1:** Demographics, lower-limb involvement and previous treatment performed in other hospitals with unsatisfactory results

24 patients	
11 male	
13 female	
Mean age at diagnosis: 6.7 ± 2.34 years (range, 4–13 years)	
6 patients with polyostotic fibrous dysplasia	
18 patients with McCune–Albright syndrome	
12 patients with monomelic involvement	
12 patients with bimelic involvement	
Closed reduction and plaster cast for femoral and tibial fractures: 9 patients	
Küntscher nailing for femoral fractures or osteotomy stabilization: 6 patients	
Peripheral plating for femoral and tibial fractures or osteotomiy stabilization: 9 patients	

We included in the study (1) patients whose femur or tibia was totally affected by FD, (2) patients who presented lower limb deformity and fracture following conservative treatment in a plaster cast, (3) patients who had lower limb deformity and fracture following surgical treatment with peripheral plates or Küntscher nails and (4) patients whose growth plates were closed. The following subjects were excluded from the study: (1) patients who had had no previous treatment; (2) patients whose femur and tibia were only partially affected by FD; (3) patients whose growth plates were still open.

At admission to our hospital, 3 patients were wheelchair users, 17 had a painful limp and 10 of them used an aid for walking, while 4 patients were fractured. Overall, the study included 34 femurs and 14 tibias. Both femoral [26] and tibial deformities and the patients’ mean age at IN are reported in Table 2.

**Table 2:** Number of cases, type of deformity before intramedullary nailing, fractures, and mean age of the patients at intramedullary nailing performed in our hospital

34 femurs:^a^	14 tibias:
• Type 1: 2 cases (1 fracture)	• Anterior bowing deformity: 2 cases
• Type 3: 1 case	• Antero-medial bowing deformity: 12 cases (1 fracture)
• Type 4: 13 cases (1 fracture)	
• Type 5: 2 cases	
• Type 6: 16 cases (1 fracture)	
Mean age at intramedullary nailing: 23.66 ± 6.06 years (range, 15–37 years)	

^a^Femoral deformities on the coronal plane were classified according to Ippolito et al. [25]

Cervicodiaphyseal titanium nails were used for femoral fixation (Unreamed Femoral Nail {UFN) with a spiral blade, Trochanteric Fixation Nail (TFN) with a helical blade and Adolescent Lateral Femoral Nail (ALFN) with cervical screws for small femurs, DePuy Synthes), while interlocking titanium nails were used for tibial fixation (Unreamed Tibial Nail (UTN) for normal-size and Unreamed Humeral Nail (UHN) for small tibias, DePuy Synthes).

The treatment of choice for PFD/MAS when the femur and tibia are totally affected by FD is IN, which must be performed starting in childhood, whether for fracture fixation or for corrective osteotomy stabilization or for chronic pain resistant to medical treatment. IN must be repeated during the child’s growth as soon as the nail becomes too short to stabilize the affected skeletal segment. Satisfactory long-term results have been reported with this management protocol [28].

The malalignment test of Paley [29] was performed on standing antero-posterior radiographs of the pelvis and lower limbs to identify the apex of the deformity and plan the level of the osteotomy. Lying radiographs were done in fractured patients and a CT scan with 3D reconstruction in 2 non-ambulatory patients. Multiapical bowing deformities [29] were prevalent and required more than one corrective osteotomy, while type 6 femoral deformities with a neck–shaft angle  ≤ 90° had a two-stage procedure [27]. Additional surgery was performed in 11 cases: 3 cases had distal varus femoral osteotomy, 6 had either femoral or tibial shortening osteotomy, whereas 2 fractured femurs in type 4 deformities had 1 additional osteotomy.

All the patients aside from the four who were fractured were evaluated clinically before IN and at follow-up using Jung et al.’s grading system [11]. In all cases, we compared the radiographs done at follow-up to those done before and after IN.

Descriptive statistics consisted of the mean ± standard deviation for parameters with normal distributions after confirmation with histograms and the Kolmogorov–Smirnov test. Comparisons among groups were performed with Student’s* t*-test, Fisher’s exact test, the chi-squared test, and the ANOVA test. Levels of significance reaching 95% or more were accepted, and a *p* of < 0.05 was considered statistically significant.

## Results

The mean age of the 24 patients at follow-up, the mean length of follow-up, and the mean scores of the patients with PFD and those with MAS at follow-up are reported in Table 3.

**Table 3:** Mean length of follow-up and mean age of the 24 patients along with their mean Jung score^a^ at follow-up

Mean length of follow-up: 9.12 ± 3.68 years (range: 4–17 years)	
Mean age at follow-up: 32.79 ± 6.54 years (range: 23–46 years)	
Monomelic polyostotic fibrous dysplasia (6 patients): 9 ± 1.2 points	
Monomelic McCune–Albright syndrome (6 patients): 6.66 ± 2.66 points	
Bimelic McCune–Albright syndrome (12 patients): 5.66 ± 4.24 points	

^a^According to the Jung et al. scoring system [10], an excellent result scores 10–9 points; good, 8–7 points; fair, 6–5 points; poor, < 5 points

The gender distribution of our patients was homogeneous, since no significant difference was observed between the numbers of male and female patients (*p* = 0.658).

Monomelic PFD patients scored significantly better than monomelic MAS patients (*p* < 0.01), while monomelic MAS patients scored better than bimelic MAS patients (*p* < 0.31). The 4 fractured patients could not be evaluated with the Jung scale before IN, while the mean Jung score of the 20 nonfractured patients was 2.52 ± 1.74 points before IN and 6.78 ± 2.23 at follow-up. The difference was statistically significant (*p* < 0.05).

**Fig. 1:**
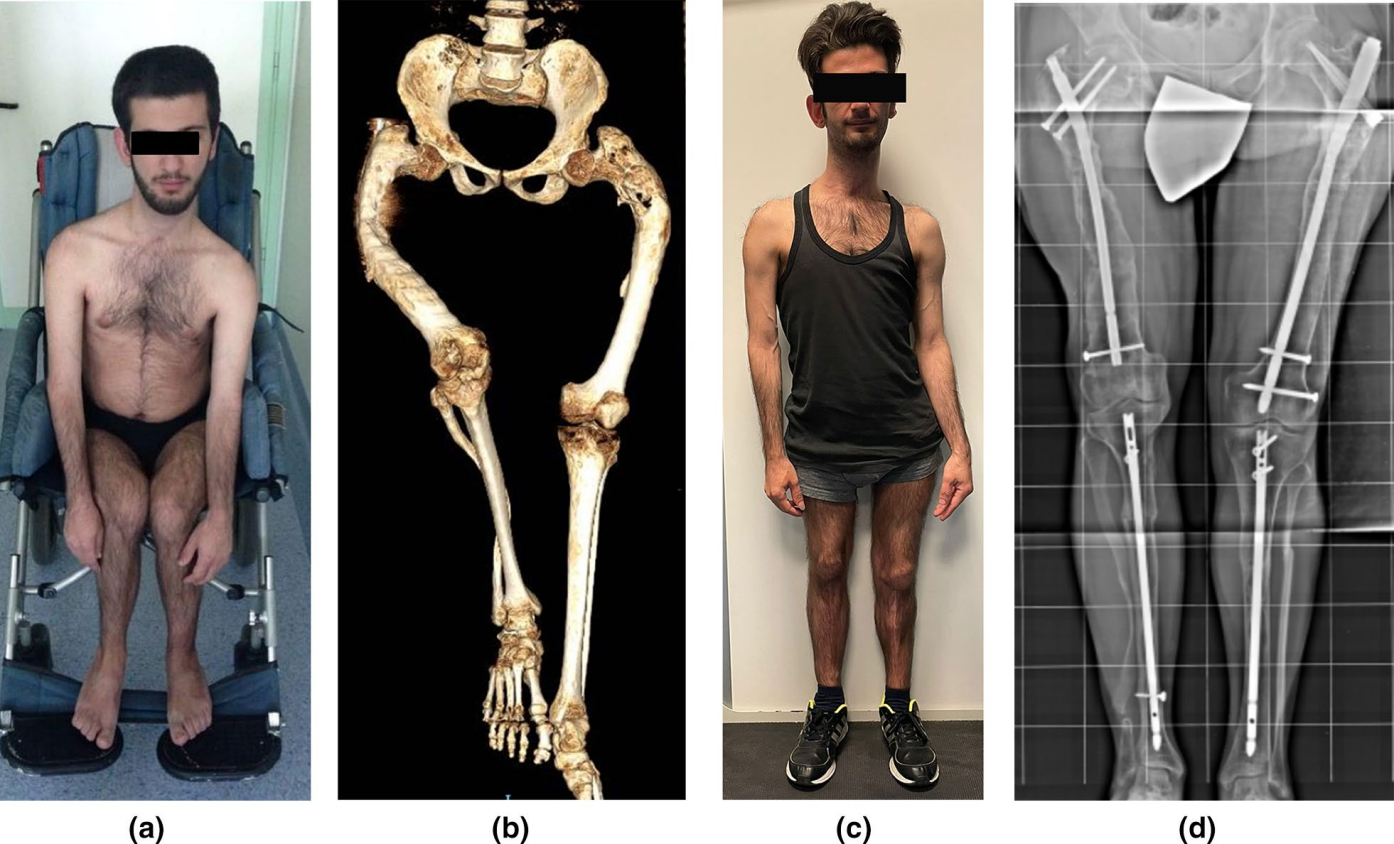
**a** Eighteen-year-old boy with McCune–Albright syndrome and bimelic fibrous dysplasia involvement. He was wheelchair dependent, with a clinical Jung score of 0 points. He had had a non-operative orthopedic treatment for several lower limb fractures. **b** Three-dimensional CT scan of the pelvis and lower limbs showing a severe bilateral shepherd’s crook deformity (type 6 femoral deformity) with 6 cm of lower limb length discrepancy. **c** At follow-up, 5 years after the last intramedullary nailing, he walked independently with a mild painless limp; he was able to perform all the activities of daily living and he had a normal social life, regardless of the 2 cm of both lower limb length discrepancy and knee height asymmetry (**d**). An excellent result was obtained, with a clinical Jung score of 9 points

**Fig. 2:**
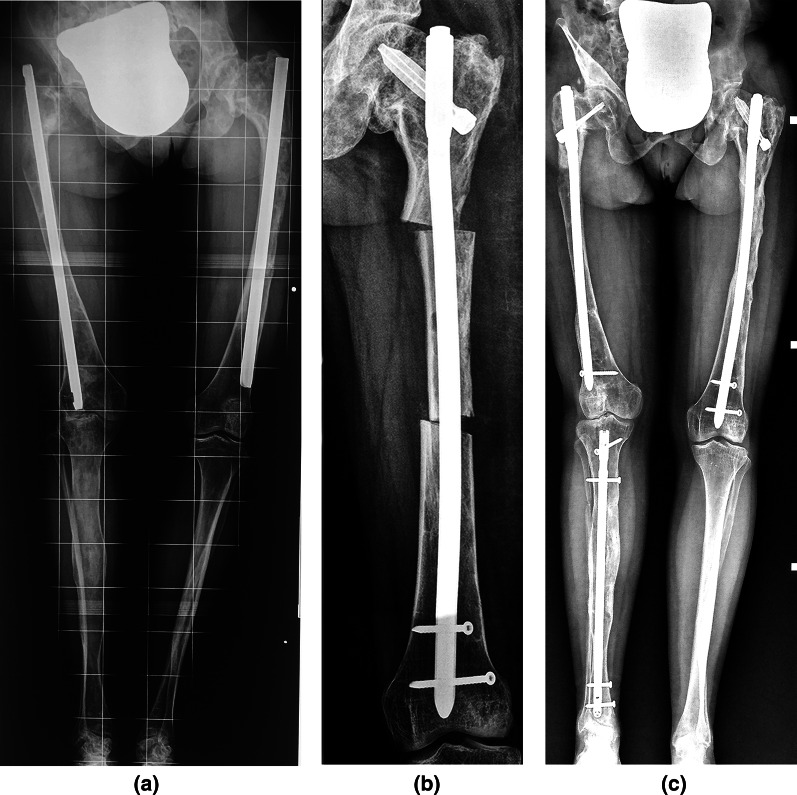
**a** Standing antero-posterior radiograph of the pelvis and lower limbs of a 22-year-old patient with McCune–Albright syndrome and bimelic fibrous dysplasia involvement, showing a bilateral shepherd’s crook deformity (type 6 femoral deformity), coxa brevis, medial bowing of the right tibia and 4 cm of lower limb length discrepancy. She had been operated on twice, with bilateral femoral Küntscher nailing performed at 10 and 17 years of age, respectively. She was able to walk with pain for only a short distance using 2 crutches, and her clinical Jung’s score was 2 points. **b** In our hospital, she had bilateral femoral and right tibial osteotomies stabilized with intramedullary nails. **c** At follow-up, 9 years later, she had 10° of residual varus deformity of the right femoral shaft, neck–shaft angles of 110° on the right and 125° on the left, 3 cm of knee height asymmetry, and 2 cm of lower limb length discrepancy compensated for by a shoe lift. She also had a slight bilateral limitation of the hip ROM, but she was able to walk independently for a long distance despite a mild painless limp. A good result was obtained, with a clinical Jung score of 8 points

Patients were pleased with the result of IN, which led to an overall improvement in their quality of life (Figs. 1 and 2). At follow-up, 11 patients had a normal gait, 10 had a mild to moderate limp, and 3 had a severe limp and used an aid for walking. Pain was still present in 7 patients when they were walking. ROM limitation was present in 11 hips with coxa brevis (Fig. 2).

**Fig. 3:**
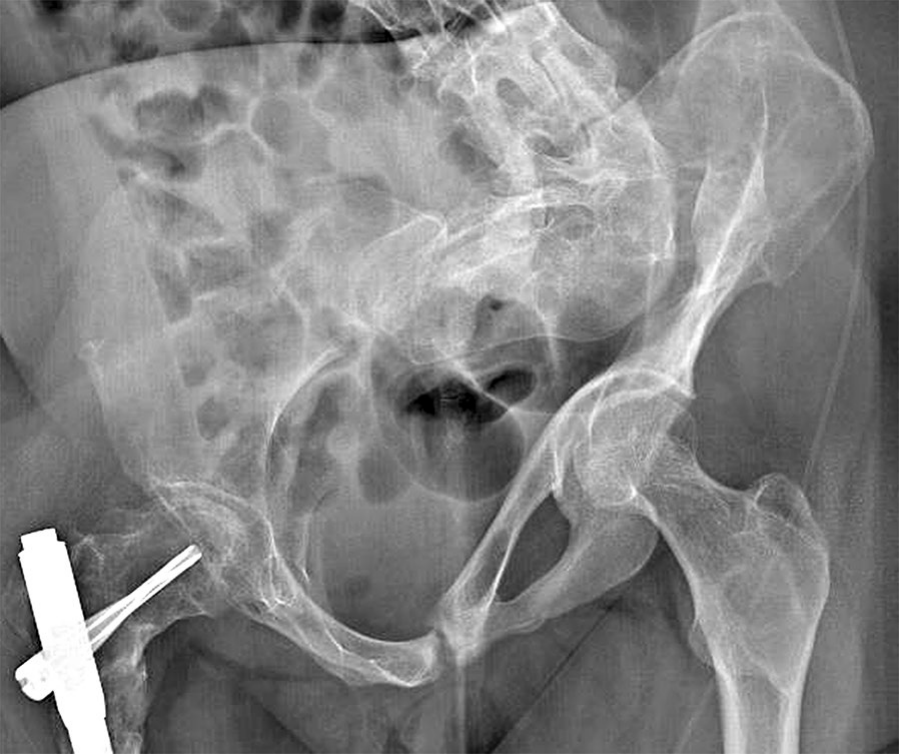
Standing antero-posterior radiograph of the pelvis and hips of a 28-year-old patient with McCune–Albright syndrome and monomelic right fibrous dysplasia involvement done at follow-up 8 years after her right femur intramedullary nailing. A 4.5 cm hip height discrepancy was present, which had increased by 3 cm during the last 4 years due to a protrusion of her left hemi-pelvis into the pelvic ring. She had a painful limp, a 3 cm shoe lift, and she used 2 crutches for walking. A poor result was obtained, with a clinical Jung score of 4 points

The following were the most important radiographic findings at follow-up. (1) Correction of the shepherd’s crook deformity was not achieved in 4 cases in which a residual varus deformity of the femoral shaft ranging from 5° to 10° was present after IN and remained unchanged at follow-up (Fig. 2). (2) In 1 MAS patient only, a recurrence of a type 6 femoral deformity with nail migration occurred 15 years after IN with full correction. She had been jogging for 2–3 km 3 times a week since the IN. (3) Eleven femurs in MAS patients lost from 5° to 25° of the neck–shaft angle correction. (4) Eight patients presented a knee height asymmetry ranging from 1 to 5 cm (Figs. 1 and 2). (5) Sixteen patients had a lower limb length discrepancy (LLD) ranging from 1 to 4 cm (Fig. 1), and 8 of them wore a shoe lift. Three of the latter had been complaining of a gradual and painful loss of the ability to walk obtained after IN, and radiographs showed spontaneous collapse of the hemi-pelvis that was extensively affected by FD along with increased LLD (Fig. 3).

Four patients had a poor result. Three of them were those in whom the hemi-pelvis had collapsed. In the fourth patient with severe groin pain and hip ROM limitation, radiographs showed advanced left hip osteoarthritis.

Two fractures occurred after IN: an undisplaced fracture of the medial femoral condyle and a pertrochanteric fracture with bending of the cervical blade caused by a high-energy trauma.

**Fig. 4:**
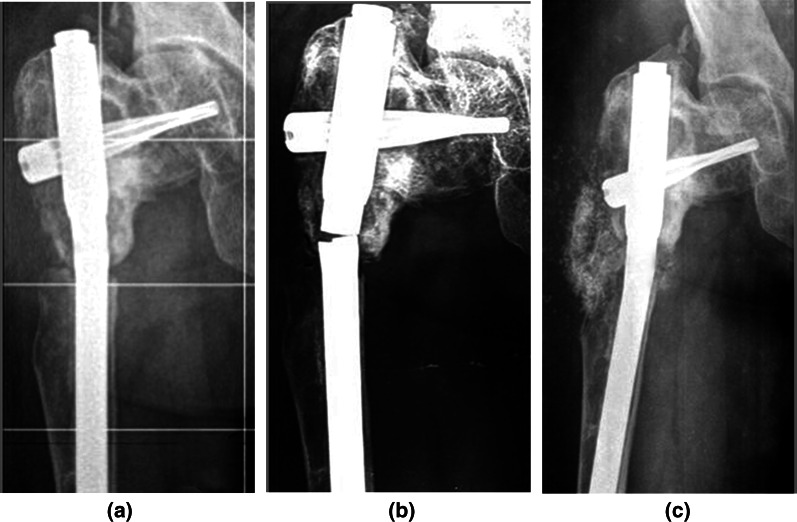
**a** Subtrochanteric asymptomatic osteotomy non-union of the right femur of the patient illustrated in Fig. 2, 1 year after the index surgery. **b** The non-union became symptomatic following the nail’s fatigue rupture. **c** The non-union healed after nail replacement and iliac crest bone grafting

Complications occurred in 10 cases. Two femoral non-unions with a broken UFN, which healed after nail replacement and autologous bone grafting (Fig. 4); 2 femoral delayed unions, which healed after the removal of the nail distal locking screws; 1 acute post-operative loss of the neck–shaft angle correction after hip valgus osteotomy, which healed after surgical revision; 3 common peroneal nerve palsies after tibial varus osteotomy, 2 of which recovered spontaneously whereas 1 required a peroneal longus tendon transfer; 1 loosened distal locking screw was removed in 2 tibial INs.

## Discussion

Management of weight-bearing bones totally affected by FD is challenging, and it may lead to patient disability when it is inappropriate, as in the patients of our cohort before IN [3, 6, 7, 12, 17]. According to our results, IN—indicated at present as the best primary treatment in long bones extensively affected by FD [3, 9–17, 26, 27]—appears to also be ideal as a salvage procedure after improper treatments.

Published expert opinions, case reports and small series with a short-term follow-up on femoral and tibial primary IN in PFD/MAS are prevalent [3, 9–16, 26]. We operated on 48 femurs and tibias of 24 patients, with an average follow-up of 9 years. As far as we know, this is one of the largest series of lower limb IN with a long-term follow-up, albeit performed after previous unsatisfactory treatments.

Our patients had had various treatments with poor results. Therefore, we can confirm that non-operative orthopedic procedures as well as femoral diaphyseal nailing without cervical stabilization must be abandoned [3, 4, 6, 9, 10, 12, 15–17]. Since peripheral plating frequently fails [3, 9–17, 26, 27], its indication should be very limited [27], although for several authors, peripheral plating still represents the preferred treatment in PFD/MAS patients with lower limb involvement [18–25].

The Zickel nail was the first cervicodiaphyseal femoral device used in PFD to prevent coxa vara thanks to its femoral neck component. On the basis of its positive use [9], validated by a follow-up of more than 20 years [3], we employed the new generation of cervicodiaphyseal nails, which also include adolescent nails for smaller femurs [30]. In the tibia, regular interlocking tibial nails were used, whereas smaller tibias were nailed with adult interlocking humeral nails.

We were able to fix all the femoral and tibial deformities except for 4 femoral type 6 deformities in which a residual varus angulation of the femoral shaft was still present after IN and remained unchanged at follow-up. Unfortunately, in those cases, the extreme softness of the fibrodysplastic bone surrounding the medullary canal drilled into the bone segments after the osteotomy did not allow the nail to maintain a straight alignment of all the femoral shaft osteotomy segments.

Only 3 validated scales are available to assess PFD patients [11, 31, 32]. Enneking’s scale has been specifically conceived for patients operated on for bone tumors [31]. Since PFD is a benign condition, we adopted the Guille et al. scale [33] as modified by Jung et al. [11]. According to the Jung scoring system—made up of 5 basic clinical parameters—almost 83% of our patients obtained satisfactory long-term results, and the clinical score for the 20 nonfractured patients significantly improved at follow-up with respect to their score before IN, which we performed after previous improper treatments.

A high incidence of fractures up to 30 years of age has been reported in MAS [5]. Therefore, fracture prevention was another important goal achieved by IN, since 83% of our patients were younger than 30 years at IN, and the 2 femoral fractures after IN could not be prevented because one occurred below the tip of the nail and the other was caused by a trauma so intense that it bent the nail cervical blade.

Both the extent of lower limb involvement and the quality of FD bone affected the result of treatment, since monomelic MAS patients fared better than bimelic ones—although the difference was not significant—and monomelic PFD patients fared significantly better than those with MAS. In the latter, the hormonal influence worsens the weakness of FD bone, as already reported [3, 5–8, 12, 17]. In this context, the loss of neck–shaft angle correction observed in 11 femurs of our MAS patients at follow-up, regardless of the support of the nail’s cervical component, may be explained by the extreme bone weakness of the upper femur.

Only 1 MAS patient had a recurrence of a type 6 femoral deformity, but she had been jogging 3 times a week for 15 years since the IN. We believe that sporting activities should be carefully selected in MAS patients with severe lower limb involvement, since IN per se is not sufficient to prevent deformity recurrence when the limb is overloaded.

Coxa brevis responsible for ROM limitation was present in several hips. It might have been caused by repeated surgery that stunted femoral neck growth, as happened in the patient illustrated in Fig. 2.

Four MAS patients ended up with an unsatisfactory result. In 3 patients with extensive pelvic involvement, one hemi-pelvis gradually collapsed, leading to a marked LLD that was compensated for with a shoe lift and an aid for walking. Severe pelvic involvement by FD was the worst risk factor for a poor result in our cohort but, as far as we know, no surgical solution has been described for this condition. The fourth patient developed severe hip osteoarthritis at 30 years of age [32]. However, surgery was not performed because the result of total hip replacement is unpredictable in PFD [34].

Lower limb length discrepancy (LLD) correction was a problem in our cohort, as already reported [3, 12, 17]. We performed shortening osteotomy, but residual LLD was still present in two-thirds of our patients, though only 8 wore a shoe lift, including the 3 with a hemi-pelvis collapse. Bone lengthening has been reported in PFD when the cortical bones are thick enough to withstand distraction [35]. Unfortunately, our cases were totally affected by FD and their cortical bones were very thin, thus not allowing lengthening [3, 17].

Knee height asymmetry was present in one-third of our patients. It was caused either by the spontaneous overgrowth of the femur or tibia—often observed in the same limb of PFD/MAS patients [2, 3, 6, 11, 12]—or by previous treatments that stunted their growth. We tried to reduce the asymmetry by performing a shortening osteotomy of the longer bone segment in cases with LLD. However, even 5 cm of residual knee asymmetry did not cause any significant limitation on the usual activities of daily living.

We had a high incidence of complications (21%). We were very surprised by the low complication rate in previous studies on primary lower limb IN; this was probably due to the small number of cases and/or to the shortness of the follow-up [4, 9–11, 13, 14, 18–25]. Zhang et al. [36] deserve particular mention for the absence of reported complications in 65 patients treated with IN during a mean follow-up of 6 years. However, we should also bear in mind that previous repeated treatments—both non-operative and surgical—might have had a negative influence on FD bone quality in several cases, causing mainly either non-union or delayed union after IN. None of the cases suffered an infection, as also previously reported [9–25, 37], with the exception of one adult PFD patient [4]. The explanation for this remains speculative.

This study has several limitations. First, it is a retrospective study with all of the corresponding possible biases. However, none of our original 24 patients were lost to follow-up. Nevertheless, a prospective study would be welcome in the future so as to avoid other possible biases. Second, our study lacks a control group. However, none of the other treatment protocols known so far for PFD/MAS are suitable for use in control groups, since the clinical histories of our patients who were previously treated with those protocols showed poor results. Moreover, an untreated control group does not make sense because it is well known that a lack of treatment generates severe disability in those cases. Third, our cohort included a small number of patients because PFD and MAS are rare diseases made rarer by our strict inclusion criteria. However, one possible solution might be to organize multicenter studies in the future by involving referral centers for those rare diseases.

In conclusion, we believe that, in PFD/MAS, IN may be considered a reliable surgical procedure to salvage previous failed treatments and to provide long-lasting satisfactory results in most treated patients.

## Data Availability

All data and material are available.
